# Robust MR-based approaches to quantifying white matter structure and structure/function alterations in Huntington's disease

**DOI:** 10.1016/j.jneumeth.2015.08.027

**Published:** 2016-05-30

**Authors:** Jessica J. Steventon, Rebecca C. Trueman, Anne E. Rosser, Derek K. Jones

**Affiliations:** a Cardiff University Brain Research Imaging Centre, School of Psychology, Cardiff University, Park Place, Cardiff CF10 3AT, UK; b Brain Repair Group, Life Science Building, 3rd Floor, School of Biosciences, Cardiff University, Museum Avenue, Cardiff CF10 3AX, UK; c Neuroscience and Mental Health Research Institute, Cardiff University, Hadyn Ellis Building, Cathays, Cardiff CF24 4HQ, UK; d School of Biomedical Sciences, Queen's Medical Centre, Nottingham University, Nottingham NG7 2UH, UK; e Institute of Psychological Medicine and Neurology, School of Medicine, Hadyn Ellis Building, Maindy Road, Cathays, Cardiff CF24 4HQ, UK

**Keywords:** CSF, cerebrospinal fluid, DTI, diffusion tensor imaging, EPI, echo-planar imaging, FWE, free water elimination, HD, Huntington's disease, MRI, magnetic resonance imaging, ROI, region-of-interest, SD, spherical deconvolution, TVF, tissue volume fraction, Diffusion MRI, Huntington's disease, Tractography, Corpus callosum, Disease burden, DTI

## Abstract

**Background:**

Huge advances have been made in understanding and addressing confounds in diffusion MRI data to quantify white matter microstructure. However, there has been a lag in applying these advances in clinical research. Some confounds are more pronounced in HD which impedes data quality and interpretability of patient-control differences. This study presents an optimised analysis pipeline and addresses specific confounds in a HD patient cohort.

**Method:**

15 HD gene-positive and 13 matched control participants were scanned on a 3T MRI system with two diffusion MRI sequences. An optimised post processing pipeline included motion, eddy current and EPI correction, rotation of the B matrix, free water elimination (FWE) and tractography analysis using an algorithm capable of reconstructing crossing fibres. The corpus callosum was examined using both a region-of-interest and a deterministic tractography approach, using both conventional diffusion tensor imaging (DTI)-based and spherical deconvolution analyses.

**Results:**

Correcting for CSF contamination significantly altered microstructural metrics and the detection of group differences. Reconstructing the corpus callosum using spherical deconvolution produced a more complete reconstruction with greater sensitivity to group differences, compared to DTI-based tractography. Tissue volume fraction (TVF) was reduced in HD participants and was more sensitive to disease burden compared to DTI metrics.

**Conclusion:**

Addressing confounds in diffusion MR data results in more valid, anatomically faithful white matter tract reconstructions with reduced within-group variance. TVF is recommended as a complementary metric, providing insight into the relationship with clinical symptoms in HD not fully captured by conventional DTI metrics.

## Highlights

• A pipeline is presented to address HD-related confounds in diffusion MRI data.
• CSF contamination affected the sensitivity to detect white matter alterations in HD.
• The choice of tracking algorithm affected the reconstruction of the corpus callosum.
• Tissue volume fraction is a complementary metric to conventional tensor metrics.
• Tissue volume fraction has increased sensitivity to clinical symptoms in HD.

## 1 Introduction

Diffusion tensor MRI, a technique to measure tissue microstructure in vivo (Basser et al., 1994), has been repeatedly applied in patients with Huntington's disease (HD) to provide evidence of white matter abnormalities both in the prodromal and symptomatic stages of disease (Dumas et al., 2012, Magnotta et al., 2009, Rosas et al., 2008). There is an evolving understanding of confounds which exist in the generation of metrics from diffusion MR images (Jones and Cercignani, 2010, Jones, 2010, Le Bihan et al., 2006, O’Donnell and Pasternak, 2014), nonetheless the emerging methodologies to address these confounds have yet to enjoy widespread adoption by the HD imaging community. The presence of these confounds, and the failure to address them, will impact on the sensitivity and specificity of the measures, and ultimately the interpretability of white matter alterations in HD, and to some extent may account for inconsistencies in the literature.

In this paper, we present an optimised processing pipeline for the analysis of diffusion MRI data which is not routinely applied, yet addresses many confounds of specific relevance in HD. Many of the optimisations presented can be performed post hoc on data already acquired; therefore there are no costs in terms of additional scan time.

### 1.1 Physiological noise

Diffusion MRI is highly sensitive to motion and physiological noise artefacts, such as those originating from participant's motion during scanning, which is a concern when imaging participants with a movement disorder. It has been shown previously that motion can produce severe artefacts which if not addressed, can result in flawed and more variable diffusion tensor metrics (Chang et al., 2005, Mangin et al., 2002, Morris et al., 2011, Walker et al., 2011, Zhou et al., 2011, Zwiers, 2010). There are methods to address physiological noise, both during the acquisition of the data and post hoc during the analysis. For example, when acquiring the diffusion-weighted image, acquisition can be restricted to periods of reduced cardiac pulsation-induced motion in order to reduce motion related to cardiac pulsation (Brockstedt et al., 1999, Chang et al., 2005, Habib et al., 2008, Habib et al., 2009, Jones and Pierpaoli, 2005). Although this approach can substantially lengthen the scan acquisition time, if the scan time is available this trade-off can result in substantially improved data quality.

In addition to off-the-shelf software packages that correct for motion and eddy-current artefacts in the image, the Robust Estimation of Tensors by Outlier Rejection (RESTORE) algorithm has been developed to identify motion-based outliers and has been shown to improve the tensor estimation on a voxel-by-voxel basis and reduce the impact of artefactual data points post hoc in diffusion MR images (Chang et al., 2005, Chang et al., 2012).

### 1.2 Contamination of the signal due to atrophy

Another confound is the presence of atrophy in the acquired diffusion MR images, which is an issue in HD, with grey and white atrophy present in HD at all disease stages (Aylward et al., 2011, Nopoulos et al., 2007, Paulsen et al., 2006, Paulsen et al., 2010, Tabrizi et al., 2011, Tabrizi et al., 2012). Cerebrospinal fluid (CSF) fills the cerebral space left by atrophied tissue; consequently contamination of MRI metrics due to CSF is highly likely in participants with HD. CSF contamination is particularly problematic in diffusion MRI (Alexander et al., 2001, Vos et al., 2011). Diffusion MRI metrics obtained at the boundaries between CSF and white matter may be contaminated, resulting in elevated measures of diffusivity, and reduced fractional anisotropy (FA) (Alexander et al., 2001). Diffusivity metrics (mean diffusivity, axial and radial diffusivity) have been shown to be more prone to errors related to CSF-contamination compared to FA, which may ultimately bias the interpretation of diffusion MRI studies (Metzler-Baddeley et al., 2012).

Techniques are available to correct for contamination, yet are not widely applied in HD research, meaning that reported abnormalities in HD may not be tissue specific and instead may include signal from CSF contamination. During data acquisition, CSF artefacts can be removed with a fluid-attenuated inversion recovery (FLAIR) sequence (e.g. Chou et al., 2005, Papadakis et al., 2002), however this is not commonly adopted due to a prohibitively long scan time and a low signal to noise ratio. More recently, a diffusion sequence utilising a shorter TR and non-zero minimum diffusion weighting was shown to reduce the effects of CSF contamination (Baron and Beaulieu, 2015).

Despite this, at present, the majority of diffusion MRI datasets will not have been acquired using such optimised diffusion sequence parameters. Instead, post hoc approaches facilitate the reduction of CSF contamination regardless of the diffusion sequence used. One such post hoc approach to correcting for CSF contamination is to fit a two compartment model to the diffusion signal, modelling one compartment as an anisotropic ‘tissue’ tensor, and the second compartment modelled as an isotropic CSF contribution to signal decay (Pierpaoli and Jones, 2004, Pasternak et al., 2009). ‘Free water elimination’ (FWE; Pasternak et al., 2009) is one such approach which can be applied post hoc to standard, single-shell diffusion MRI data which is advantageous for clinical studies where scans are time limited. This approach produces corrected values of tensor metrics (FA and diffusivity metrics MD, AD and RD) which have been shown to be more specific to tissue alterations compared to non-corrected tensor metrics (Metzler-Baddeley et al., 2012). An additional advantage is that the approach can explicitly estimate, on a voxel-by-voxel basis, the ‘tissue volume fraction’ (TVF), which is the fraction of signal arising from the tissue compartment (Pasternak et al., 2009).

### 1.3 Sub-optimal tract reconstruction approaches

Diffusion tractography is an analysis approach which enables specific white matter pathways to be reconstructed in their entirety in 3 dimensions (Basser et al., 2000, Conturo et al., 1999, Jones et al., 1999a, Jones, 2008, Mori et al., 1999). To date, only a handful of studies (Bohanna et al., 2011, Phillips et al., 2013, Phillips et al., 2014, Poudel et al., 2014) have applied diffusion tractography analysis in HD. A conventional tractography approach is diffusion tensor imaging (DTI)-based tractography, in which the directional information from the diffusion tensor is used, with streamlines following the principal eigenvector (Basser et al., 2000). The diffusion tensor model is limited in areas of complex fibre architecture as only a single fibre orientation can be modelled, which means that the model is inadequate in areas of crossing, diverging, or converging white matter tracts (Alexander et al., 2001, Tuch et al., 2002, Wedeen et al., 2005). More advanced approaches have been developed but have yet to be applied in HD. In this work, we apply spherical deconvolution as the tracking algorithm (Alexander, 2005, Dell’Acqua et al., 2010, Tournier et al., 2007), but we also acquire the diffusion MR data with a larger number of unique gradients in order to improve the diffusion profile (Alexander, 2005, Frank, 2002, Tuch et al., 2002) and overcome the well-established limitations of the diffusion tensor model.

### 1.4 Non-specific tensor-based metrics

An additional issue in diffusion MR analysis is the reliance on conventional tensor-based metrics which are highly sensitive to pathology but limited in terms of the specificity to underlying pathophysiology, presenting a challenge for the interpretation of results. TVF, which is the output of the FWE approach to correct for CSF-related contamination (detailed above), has potential as a more specific outcome measure in diffusion MRI studies. The TVF is expected to decrease in the presence of neuroinflammation (Wang et al., 2011) and has been shown to be sufficiently sensitive to detect changes in patients with first-episode schizophrenia (Pasternak et al., 2012), brain injury (Pasternak et al., 2014) and Parkinson's disease (Ofori et al., 2015).

Complementary MRI metrics may provide new insights into the role of white matter in HD pathology. At present, it is known that mutant Huntingtin (HTT) aggregates localise in the axons as well as in neurons (Li et al., 2001, Li, 1999, Sinadinos et al., 2009), with evidence to show disrupted axonal transport in HD (Li et al., 2001, Li and Conforti, 2013, Sinadinos et al., 2009). Myelin breakdown may also contribute to white matter atrophy in HD (Bartzokis et al., 2007) with a number of indirect research findings implicating myelin, such as thinner myelin sheaths possibly resulting from impaired cholesterol metabolism in HD (Dietschy and Turley, 2004, Saher et al., 2005, Valenza and Cattaneo, 2010, Valenza et al., 2005, Valenza et al., 2007, Xiang et al., 2011) and decreased expression of myelin-based protein in mouse models of HD (Xiang et al., 2011).

In summary, in this study we present an optimised acquisition and analysis pipeline that addresses a number of confounds which can affect the sensitivity and specificity of diffusion MRI analysis in HD. Our pipeline includes a number of processing steps not routinely applied, including: (1) motion, eddy-current and EPI correction with corresponding rotation of the encoding vectors, (2) correction for CSF-related contamination of the diffusion signal using free water mapping, (3) an optimised spherical deconvolution based approach to tractography in order to reconstruct crossing fibres, (4) application of a complementary diffusion MRI-derived metric which is shown to be more sensitive to clinical markers of HD compared to conventional tensor-based metrics. To demonstrate the results of the pipeline, we have chosen to examine the corpus callosum as the majority of callosal fibres arise from neocortical pyramidal cells (Le Bé et al., 2007) which are known to be affected early on in the disease course of HD (Sach et al., 2004). The corpus callosum has been shown to be thinner in HD participants compared to controls (Di Paola et al., 2012, Rosas et al., 2011, Vonsattel et al., 1985) and diffusion MRI studies have found reduced FA and/or increased MD in the corpus callosum in HD participants (Bohanna et al., 2011, Di Paola et al., 2012, Di Paola et al., 2014, Dumas et al., 2012, Kincses et al., 2013, Müller et al., 2011, Poudel et al., 2015, Rosas et al., 2010). Importantly, the corpus callosum is a fibre pathway prone to contamination by CSF due to the proximity to the lateral ventricles.

## 2 Materials and methods

### 2.1 Participants

The study was conducted with ethical approval from South West Wales Research Ethics Committee. 15 HD gene-positive participants were recruited from the South Wales HD clinic, based at Cardiff University, along with 13 healthy age, gender and education-matched controls. Independent t-tests found no difference in age between the HD and control group, *t*(26) = 0.33, *p* > 0.05; demographic information is shown in Table 1. Clinical assessment was performed by an experienced neurologist using the Unified Huntington's Disease Rating Scale (UHDRS). Patients were classified as pre-symptomatic (*n* = 10) or early symptomatic (*n* = 5) on the basis of their UHDRS motor and UHDRS Total Functional Scale (TFC) scores.

Matching for educational attainment was conducted based on research showing that in comparison to people with low educational attainment, people with higher educational attainment have delayed symptom onset, or equivalent cognitive impairment despite a greater degree of neuropathology (Stern, 2009).

### 2.2 Clinical measures

As a measure of motor speed, the Speeded Tapping Test (Reitan, 1979) was used. This is a computerised test of finger tapping speed that requires a participant to press a button on a keyboard as quickly as possible. Participants were instructed to use the index finger of their dominant hand and instructions were provided to ensure consistency in the position of the hand. The outcome measure was the average tapping rate over 3 trials and the test was conducted on the day of the MRI acquisition, prior to scanning.

In addition, HD participants had been examined on the Unified Huntington's Disease Rating Scale (UHDRS) in the 6 month period prior to scanning (as part of a separate observation study), thus the UHDRS Motor score and the UHDRS Total Functional Capacity (TFC) score were available. The TFC scale (Shoulson and Fahn, 1979) provides a general measure of functioning not restricted to the motor domain and is the main assessment tool of functional status in HD clinical care and research, originally designed to assess progression of HD in symptomatic patients with an emphasis on self-care, mobility and independence.

Disease burden was calculated according to the previously described formula (age × [CAG − 35.5]), where CAG is the number of CAG repeats (Penney et al., 1997) and is a presumed index of the cumulative toxicity of mutant Huntingtin.

### 2.3 MRI acquisition

Diffusion MRI images were acquired on a 3T HDx Signa MRI system (General Electric). Two separate diffusion-weighted MRI acquisitions were collected: a sequence optimised for diffusion tensor imaging (DTI) and an additional sequence more suited to recovery of fibre orientations through spherical deconvolution approaches. Both sequences were peripherally gated to the cardiac cycle to reduce artefacts related to cardiac pulsation. Parallel imaging [ASSET factor = 2] was used to reduce the effects of susceptibility by reducing the echo train duration. For both diffusion-weighted sequences, a twice-refocused spin-echo echo-planar imaging (EPI) sequence was used which provided whole axial oblique brain coverage; gradient onset times and diffusion times (*δ*) are shown in the Supplementary information. For both sequences, data were acquired from 60 axial slices with a resolution of 1.8 × 1.8 × 2.4 mm and an acquisition matrix of 96 × 96. Cardiac gating resulted in *a* variable TR for individual participants. For the sequence optimised for DTI, the *b*-value was 1000 s/mm^2^, the TE was 84.6 ms and data were acquired with diffusion encoded along 30 isotropic gradient directions with 3 non-diffusion-weighted images acquired. For the second diffusion MR sequence, the *b*-value was 2000 s/mm^2^, TE was 97.3 ms and data was acquired with diffusion encoded along 45 isotropically-distributed (Jones et al., 1999b) gradient directions with 3 non-diffusion-weighted images acquired. A higher *b* value was employed because higher *b* values have been shown to give better angular resolution (Alexander et al., 2001). The gradient directions used were optimally re-ordered using the approach proposed by Cook et al. (2007) and using the Camino software package (Cook et al., 2006) so that if the scan was stopped before completion, the measurements already taken would be uniformly distributed in the sampling space.

### 2.4 Image pre-processing

Diffusion weighted images were analysed using Explore DTI version 8.3 (Leemans et al., 2009).

#### 2.4.1 Correction for motion, eddy currents and field inhomogeneity

Images were first corrected for distortions due to motion, eddy currents and field inhomogeneity related to the echo-planar imaging (EPI) sequence. EPI is the most common readout strategy for diffusion MRI acquisition but is inherently sensitive to a number of different confounds (e.g. off-resonance, susceptibility and eddy current effects, see Jones and Cercignani, 2010, for a review). EPI-related field inhomogeneities were corrected for using the approach of Wu et al. (2008); each diffusion MR image was registered to the T_1_-weighted image using a non-rigid transformation with the FA map as the reference image. Warps were computed using elastix (Klein et al., 2010) using normalised mutual information as the cost function and constraining deformations to the phase-encoding direction. Thus, the diffusion-weighted MR images were brought into the same undistorted space as the T_1_-weighted structural image and all registrations were visually inspected for accuracy. After performing the correction, any rotations that were applied to the diffusion-weighted volumes were then also applied to the encoding vector (Leemans and Jones, 2009) and the signal intensity was modulated by the Jacobian determinant of the transformation (Jones and Cercignani, 2010), both processing steps that are commonly overlooked in many studies (not just in HD studies). The data were re-inspected in three orthogonal planes after performing the correction (Jones and Leemans, 2011).

#### 2.4.2 Estimation of the diffusion tensor

A modified version of the robust estimation of tensors by outlier rejection (RESTORE) algorithm was used to estimate the tensor and remove outliers (Chang et al., 2005), with the Walker method (Walker et al., 2011) used for estimating the standard deviation of the background noise, as implemented in Chang et al. (2012). Artefactual data points were identified by robust fitting and excluded on a voxel-by-voxel basis (see Supplementary information). A visual inspection of the residuals to the tensor fit for each diffusion-weighted image was then performed in three orthogonal planes (Jones and Leemans, 2011, Tournier et al., 2011).

#### 2.4.3 Free water elimination

The images were then corrected to account for contamination due to free water using the free water elimination (FWE) approach (Pasternak et al., 2009). Corrected tensor maps were produced along with tissue volume fraction maps.

#### 2.4.4 Diffusion tractography analysis

Given that the majority (∼90%) of white matter voxels contain multiple fibre orientations (Jeurissen et al., 2013) spherical deconvolution based tracking algorithms were employed on the *b* = 2000 s/mm^2^ images in order to achieve a more reliable reconstruction (Alexander, 2005, Tuch et al., 2002) while DTI indices were calculated from the co-registered *b* = 1000 s/mm^2^ images. Whole-brain tractography was performed using the damped Richardson–Lucy algorithm (Dell’Acqua et al., 2010), a modified spherical deconvolution (SD) method shown to be robust to spurious peaks in the fibre orientation distribution (FOD) which assumes a diffusion tensor model when obtaining the diffusion profile, and uses adaptive regularisation which includes an isotropic term to model partial volume. For comparison purposes, a diffusion tensor-based tractography analysis was also conducted (Basser et al., 2000) with a FA threshold of 0.2. For both approaches, seed points were arranged in a 2 × 2 × 2 mm grid in white matter and fibre pathways were traced through the data for fibres greater than 5 mm in length and less than 500 mm in length, propagating in 0.5 mm steps, with an angle threshold of 45°. Parameters specific to the SD algorithm were: *α* = 1, algorithm iteration = 400, *η* = 0.06 and *ν* = 8 as regularisation terms (see Dell’Acqua et al. (2010) for full details of these parameters).

**Fig. 1:**
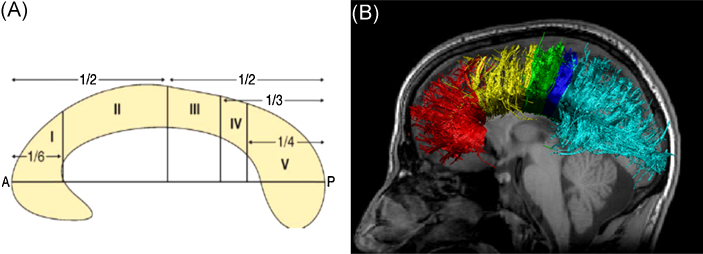
(A) Schematic of corpus callosum parcellation adapted from Hofer and Frahm (2006). (B) Transcallosal fibre tracts from a single male healthy control subject overlaid onto native T_1_ reference image. Sagittal view of reconstructed fibres projecting into the prefrontal lobe (red), premotor and supplementary motor areas (yellow), primary motor cortex (green), primary sensory cortex (dark blue), parietal, occipital and temporal lobe (light blue). (For interpretation of the references to colour in this figure legend, the reader is referred to the web version of this article.)

The corpus callosum was reconstructed using the Hofer and Frahm (2006) approach (Fig. 1) which uses 5 vertical subdivisions to parcellate the corpus callosum in accordance with defined cortical connections. Briefly, a geometric baseline was established in the mid-sagittal slice by connecting the most anterior and posterior points of the corpus callosum. Seed points were drawn on this mid-sagittal slice, and after visual inspection, spurious fibres not travelling to the cortical region were removed. Spurious fibres were removed using ‘NOT’ gates, which remove all fibres passing through the region-of-interest in order to isolate the pathway.

#### 2.4.5 Region-of-interest parcellation analysis

For the 2-dimensional region-of-interest (ROI) analysis, ROIs were derived from the ICBM-DTI-81 white-matter atlas provided by John Hopkins University (Mori et al., 2005). The FA map was non-linearly registered to the ICBM FA template image (Andersson et al., 2010) and this warp was then applied to the MD, AD, RD and TVF map. A mask was created for each ROI and the mean value in each ROI was obtained.

### 2.5 Statistical analysis

All descriptive data are presented as mean ± standard error of the mean. Basic descriptive statistics and statistical analyses were performed using SPSS Statistics (IBM, version 20). Outlier profiles were first examined and values over 3 standard deviations from the mean were deemed to be extreme outlier values and were excluded case-wise. Statistics were corrected for multiple comparisons using the classical one-stage false discovery rate (FDR) method (Benjamini and Hochberg, 1995) at 5%. For the analysis of the diffusion tensor metrics, FA and MD values were principally examined. Where significant main effects or interactions were found, post hoc analyses of the component eigenvalues, axial diffusivity (AD; *λ*1) and radial diffusivity (RD; [*λ*2 + *λ*3]/2), were conducted. Where age was found to significantly correlate with any MRI measure, age was added as a covariate in the analysis. As the distribution of many of the clinical metrics is likely to be skewed due to sampling bias inherent in the study (e.g. cognitive capacity to consent, minimal chorea for neuroimaging purposes), the Shapiro–Wilk test was used to formally test normality at *α* = 0.01 before testing the relationship with MRI measures. Where the assumption of sphericity was violated, the Greenhouse–Geisser correction (Greenhouse and Geisser, 1959) was used which alters the degrees of freedom and produces an *F*-ratio where the Type I error rate is reduced. Spearman's Rank-Order correlational analysis was used for non-normal distributions.

## 3 Results

### 3.1 DTI based tractography produces incomplete tract reconstructions

**Fig. 2:**
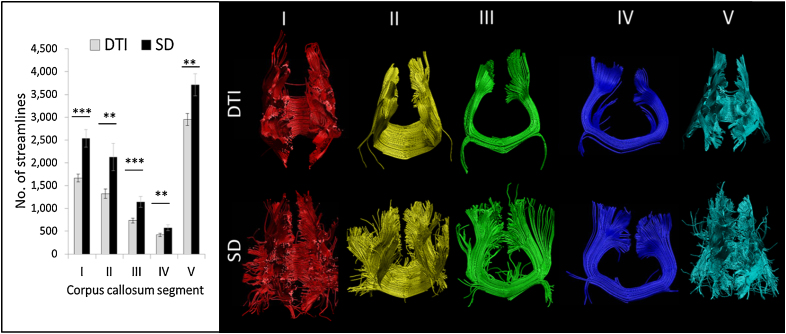
The number of streamlines reconstructed for each segment of the corpus callosum was significantly reduced with the conventional diffusion tensor-based approach (DTI; top row) compared to a spherical deconvolution (SD) based approach (bottom row). Tracts displayed in the coronal plane (segment IV and V are displayed in the reverse coronal plane for visualisation purposes) for a single representative male participant with HD. ***p < 0.001 FDR-adjusted, **p < 0.01 FDR-adjusted.

One dataset from a HD participant was not included in the tractography analysis due to poor tract reconstructions in the presence of marked atrophy. In addition, it was not possible to reconstruct the corpus callosum in an anatomically valid manner using the DTI approach for a further HD participant. For tracts reconstructed with DTI-based and SD-based tractography, the number of streamlines reconstructed for each segment of the corpus callosum was examined. There was a significant interaction between callosal segment and tractography approach (*F*_2.61,59.98_ = 6.99, *p* < 0.001, *ɛ* = 0.652); examining the 5 segments individually revealed that SD-tractography produced significantly more streamlines in every callosal segment compared to DTI-based tractography (Fig. 2), all *p* < 0.01 FDR-adjusted. The SD-approach produced a more entire reconstruction of the corpus callosum which included more lateral fibres.

### 3.2 DTI indices are contaminated by CSF in the corpus callosum

#### 3.2.1 Tractography analyses

**Fig. 3:**
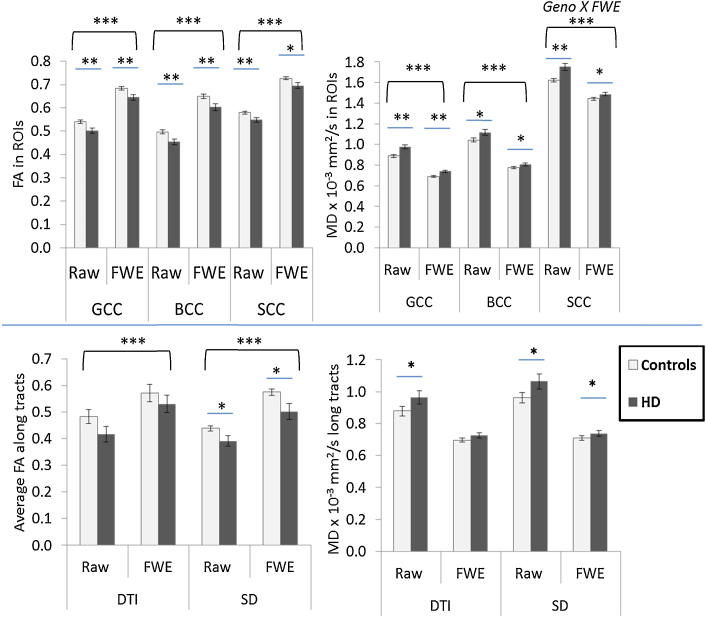
Tensor metrics FA and MD values in the corpus callosum. Top panel shows values obtained using a region-of-interest (ROI) based approach. Bottom panel shows values obtained using two tractography algorithms, DTI and SD. Raw values shown are before free water elimination (FWE). Error bars = standard errors of the mean. GCC: genu of corpus callosum, BCC: body of corpus callosum, SCC: splenium of corpus callosum, DTI: diffusion tensor imaging, FA: fractional anisotropy, SD: spherical deconvolution. ***p < 0.001, **p < 0.01, *p < 0.05, all FDR-adjusted. *Geno X FWE* refers to the statistical interaction between gene status and FWE.

To be comparable to previous studies in HD which have used DTI-based tractography, analysis was performed on tractography results using both the conventional DTI model and the more advanced spherical deconvolution tractography algorithm. A comparison of the groups (HD vs. control) and the two tracking approaches was conducted; FA and MD values obtained in the corpus callosum using both approaches are shown in Fig. 3. For differences between HD and control participants, FA values were reduced by 3–17% and MD values were increased by 2–17% in HD participants compared to healthy controls, depending on the choice of tracking algorithm, the corpus callosum segment, and whether partial volume correction had been performed. Age was found to significantly correlate with FA and diffusivity metrics in many of the callosal segments, and was thus added as a covariate in all analyses. FA values were significantly lower in HD participants compared to controls in tracts reconstructed with the SD algorithm both before and after correction (uncorrected: *F*_1,19_ = 11.37, FWE: *F*_1,20_ = 9.21, both *p* < 0.05 FDR-corrected) whereas there were no group differences in FA values for tracts reconstructed with the DTI algorithm both before and after correction (both *p* > 0.05 FDR-corrected). MD values were significantly higher in HD participants compared to controls when analysed with both DTI and SD tractography, however MD values were only significantly higher for tracts reconstructed with DTI prior to FWE (*F*_1,21_ = 8.25, *p* < 0.05 FDR-corrected), with no group differences in MD values after FWE (*F*_1,20_ = 6.91, *p* > 0.05), as shown in Fig. 3.

Post hoc analyses of the component eigenvalues AD and RD revealed significantly elevated RD in HD participants compared to controls for tracts reconstructed with DRL (uncorrected: *F*_1,20_ = 8.19, FWE: *F*_1,20_ = 13.90, both *p* < 0.05 FDR-corrected) and for uncorrected tracts (before FWE) reconstructed with DTI (*F*_1,21_ = 6.62, *p* < 0.05 FDR-corrected). This discrepancy between uncorrected and FWE results demonstrates that free water in the imaging voxel can confound tissue microstructural measures if CSF-partial volume contamination is not addressed. The results of post hoc tests for each segment of the corpus callosum are shown in Supplementary Table 2.

When comparing DTI indices before and after FWE, FA values in the corpus callosum were significantly higher (25–30%) after FWE (*F*_1,19_ = 366.9, *p* < 0.001) whilst MD values were significantly reduced (23–29%) in the corpus callosum after FWE (*F*_1,20_ = 4.73, *p*< 0.05). Similarly, AD and RD values were significantly reduced in the corpus callosum after FWE (AD: 11–15%; mean AD values (×10^−3^ mm^2^/s): DTI uncorrected = 1.41 ± 0.02, DTI FWE = 1.25 ± 0.02, DRL uncorrected = 1.44 ± 0.03, DRL FWE = 1.23 ± 0.02; RD: 33–34%, mean RD values (×10^−3^ mm^2^/s): DTI uncorrected = 0.70 ± 0.035, DTI FWE = 0.46 ± 0.02, DRL uncorrected = 0.75 ± 0.03, DRL FWE = 0.49 ± 0.02; *F*_1,19_ = 4.61 and 8.55 respectively, both *p* < 0.05 FDR-corrected). For FA, MD, AD and RD values, there was no interaction between FWE and the tractography algorithm used (all *p* > 0.05) or between FWE and the corpus callosum segment (all *p* > 0.05). Taken together with the above results, this suggests that partial volume contamination contributes to tensor metrics in tractography regardless of the tractography algorithm used.

The effect of FWE interacted with gene status (HD vs. healthy controls) for MD and AD values, however this did not survive FDR-correction (adjusted *p*-value = 0.066). The effect of FWE did not interact with age, callosal segment or tractography algorithm for any of the DTI metrics (all *p* > 0.05).

### 3.3 ROI analyses

As an alternative to the tractography analysis, we then compared the corrected tensor metrics and the mean tissue volume values in white matter ROIs in the corpus callosum (genu, body and splenium of the corpus callosum: GCC, BCC and SCC respectively). Age was again correlated with many of the diffusion metrics and was added as a covariate. 2 outliers (>3 SD) were removed from the analysis in the body of the corpus callosum; both were cases from HD participants. As with the tractography analyses, FA values were significantly reduced in HD participants compared to controls (4–9%, *F*_1,25_ = 10.53, *p* < 0.01 FDR-corrected). MD values were 3–9% higher in HD participants compared to controls (see Fig. 3 for mean values in each ROI), and an interaction was found between gene status and FWE for all diffusivity values (*F*_1,25_ = 9.981, 8.73, and 8.663 for MD, AD, and RD respectively, *p* < 0.01 FDR-corrected). Unlike the tractography analysis, there was a significant interaction between FWE and age for all diffusivity values (*F*_1,25_ = 7.75, 7.14, and 13.80 for MD, AD, and RD respectively, *p* < 0.05 FDR-corrected). Post hoc analyses showed a main effect of FWE for control participants (*p* < 0.01 FDR-corrected) whereas for HD participants, there was a significant interaction between age and FWE (*p* < 0.05 FDR-corrected) suggesting that CSF-related contamination of the diffusion signal affects HD and control participants differently. The results of post hoc analyses in individual segments are shown in Supplementary Table 2; *q*-values (FDR-corrected *p*-values) increased (i.e. became less significant) for FA and MD values after FWE, and suggest that failure to correct for CSF contamination can result in macrostructural effects being interpreted as a change in tissue microstructure.

An interaction was found between the FWE and the callosal ROI (*p* < 0.01 FDR-corrected) for MD values; whereas FWE had a main effect on MD values in the genu and body of the corpus callosum, there was instead an interaction between age and FWE (*F*_1,25_ = 4.65, *p* < 0.05); age was more highly correlated with uncorrected MD values (*r* = 0.35, *p* > 0.05) compared to MD values after FWE (*r* = 0.21, *p* > 0.05).

### 3.4 TVF is a clinically relevant metric in HD

Age was found to significantly correlate with TVF obtained along the tracts and averaged across the callosal ROIs in many of the tract segments/ROIs and was thus added as a covariate in the analyses. For the ROI analysis, tissue volume fraction (TVF) was significantly reduced in HD participants compared to controls, *F*_1,25_ = 13.00, *p* < 0.001. A main effect of callosal ROI was found (*F*_2,50_ = 4.15, *p* < 0.05). Post hoc tests on the individual ROIs showed that TVF was significantly reduced in the genu (HD = 0.62 ± 0.0090, control = 0.66 ± 0.0072, *F*_1,25_ = 11.78, *p* < 0.01 FDR-corrected), body of the corpus callosum (HD = 0.57 ± 0.0106, control = 0.60 ± 0.0068, *F*_1,25_ = 6.56, *p* < 0.05 FDR-corrected) and the splenium (HD = 0.62 ± 0.0109, control = 0.66 ± 0.0062, *F*_1,25_ = 14.00, *p* < 0.01 FDR-corrected). For TVF along the corpus callosum reconstructed with tractography, there was a significant interaction between genotype and corpus callosum segment for TVF (*F*_4,76_ = 3.112, *p* < 0.05), whereas the choice of tractography algorithm had no effect on the TVF (*p* > 0.05). Post hoc analyses in individual segments showed that TVF was reduced in HD participants compared to controls (see Supplementary Table 3 for mean values) however this difference only survived correction for multiple comparisons in the most anterior corpus callosum segment, which contains fibres projecting into the prefrontal lobe (*F*_1,23_ = 10.565, *p* < 0.05).

**Fig. 4:**
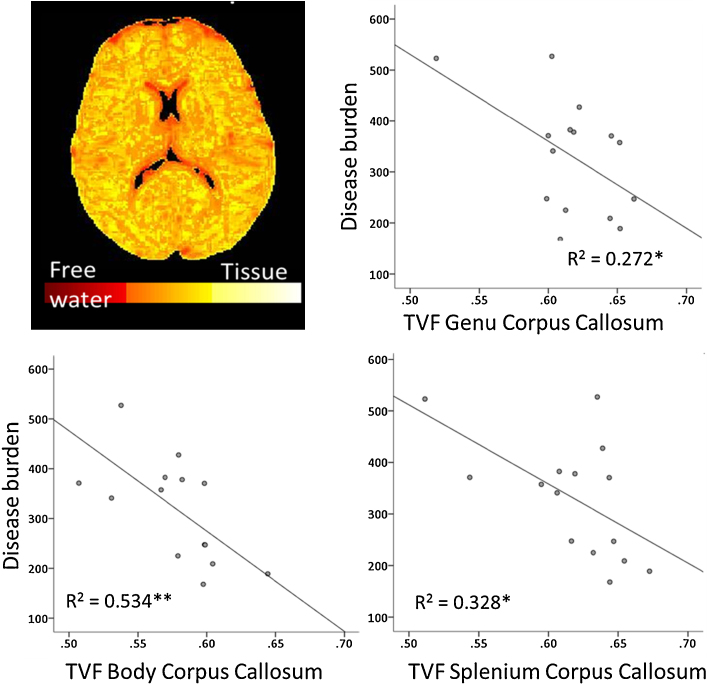
Relationship between disease burden and tissue volume fraction (TVF) in the 3 corpus callosum ROIs with linear fit line overlaid. **p < 0.01 FDR-corrected, *p* < 0.05 FDR-corrected.

Disease burden, determined by CAG repeat length and age, was normally distributed (Shapiro–Wilks = 0.935, df = 13, *p* > 0.05) and also benefits from not being affected by clinical judgement. TVF in the corpus callosum ROIs was found to be negatively correlated with disease burden in the genu (*r* = −0.521, *p* < 0.05 FDR-corrected), body (*r* = −0.731, *p* < 0.01 FDR-corrected) and the splenium (*r* = −0.572, *p* < 0.05 FDR-corrected) such that a higher disease burden was associated with reduced tissue volume in the corpus callosum, with the strongest correlation found in the body of the corpus callosum (see Fig. 4). Similarly, in the tractography analysis, a relationship between TVF and disease burden was found in the third segment of the corpus callosum which contains inter-hemispheric connections between the primary motor cortices, although this did not survive correction for multiple comparisons (*r* = −0.588, *p* < 0.05 uncorrected). In comparison, FA values obtained in both the ROI and tractography analyses were not significantly correlated with disease burden (all *p* > 0.05, Supplementary Fig. 2). Increased disease burden was associated with increased MD in the callosal body ROI (*r* = 0.571; Supplementary Fig. 2) and 4th segment of the corpus callosum (*r* = 0.575) in the tractography analysis although these did not survive multiple comparison tests (adjusted *p* value > 0.05). This suggests that TVF is a more sensitive index of disease burden compared to conventional DTI metrics.

Finger tapping speed, a measure of motor function, was found to be significantly reduced in HD participants compared to healthy controls (*F*_1,25_ = 7.87, *p* < 0.05), and was normally distributed in both HD and control participants (Shapiro–Wilks = 0.944 and 0.865, df = 15 and 13 respectively, both *p* > 0.01). For diffusion tensor metrics FA and MD, there was no relationship with finger tapping speed for control participants (all *p* > 0.05) whereas FA values along the tracts of the 3rd segment of the corpus callosum were correlated with finger tapping speed in HD participants only (*r* = 0.80, *p* < 0.05 FDR-corrected). Post hoc analyses revealed that in HD participants, the average RD value along the tracts of the 3rd segment of the corpus callosum was also correlated with finger tapping speed (*r* = −0.801, *p* < 0.05 FDR-corrected). In the ROI analysis, average FA and MD were not significantly correlated with finger tapping speed in control or HD participants (all *p* > 0.05). When examining the relationship between motor speed and TVF, a positive correlation was found between motor speed and TVF in the tracts of the 5th segment of the corpus callosum averaged across the entire corpus callosum in HD participants (*r* = 0.86, *p* < 0.05). The same relationship was not found in control participants, *r* = −0.040, *p* > 0.05, suggesting that TVF is sensitive specifically to clinical markers in HD.

In comparison, the UHDRS motor score (assessed in the 6 months prior to the MRI session) was correlated with TVF in the body and splenium ROI of the corpus callosum (*r* = −.667 and −.636 respectively, *p* < 0.05 FDR-corrected). The UHDRS motor score was also significantly correlated with average FA (*r* = −0.759, *p* < 0.05 FDR-corrected) average MD (*r* = 0.766, *p* < 0.05 FDR-corrected) and average RD (*r* = 0.793, *p* < 0.05 FDR-corrected, post hoc analysis) along the 3rd segment of the corpus callosum, which contains inter-hemispheric connections to the primary motor cortices, and with average MD values (*r* = 0.725, *p* < 0.05 FDR-corrected) and RD values (*r* = 0.764, *p* < 0. 05 FDR-corrected, post hoc analysis) along the 4th segment of the corpus callosum which contains connections to the primary sensory cortices. FA, MD, and TVF values were not significantly correlated with the non-normally distributed UHDRS Total Functional Capacity (TFC; Shapiro–Wilk = 0.877, *p* < 0.01, mean = 11.67, SD = 2.35, range = 7–13) after adjusting for age.

## 4 Discussion

A number of confounds have been identified in the analysis and subsequent interpretation of diffusion MRI data which are particularly pertinent to Huntington's disease (HD). In this study, we apply techniques not routinely applied in HD studies to address a number of these confounds with an optimised acquisition and post processing protocol. In the corpus callosum, the largest white matter pathway in the brain and the tract that has received the most attention in the HD literature, we show that the diffusion metrics are contaminated by CSF partial volume in both a 2-dimensional region-of-interest (ROI) analysis and a diffusion tractography analysis. This contamination suppresses FA values and inflates MD values which change the statistical outcome and ultimately the interpretation of HD-related changes. We applied a tractography algorithm capable of resolving multiple fibre orientations, and found that a more complete reconstruction of the corpus callosum was achieved compared to the conventional yet limited diffusion tensor-based approach, improving the anatomical validity of the results with increased sensitivity to detect group differences. Finally, for the first time in HD, we examined tissue volume fraction (TVF), which is the estimated fractional volume of tissue after free water elimination (FWE), and found that it is reduced in the corpus callosum of HD participants, and is sensitive to both disease burden and motor speed in HD participants, and in some instances is more sensitive than tensor metrics.

In line with previous work, white matter microstructural abnormalities were found in the corpus callosum in HD participants. The TVF was found to differ in the three callosal ROIs and in the callosal segments reconstructed with tractography; an interaction was found between genotype and callosal segment, with reduced TVF found in HD participants in the most anterior corpus callosum segment, which contains fibres projecting into the prefrontal lobe. These regional differences in free water suggest that more global correction approaches (e.g. correcting for whole brain volume) will not be sufficient (Metzler-Baddeley et al., 2012). In the ROI analysis, group based differences in the tensor metrics become less pronounced after FWE, suggesting that differences in tissue macrostructure also exist in these ROIs, although the statistical outcome was not affected. In contrast, for tractography analyses, group based differences in diffusivity become more pronounced after FWE, with intra-group variance reduced. Thus corrected tensor metrics were more sensitive to differences between HD and control participants, with improved specificity to examine microstructural changes, rather than a combination of tissue macrostructure and microstructure, as is the case when CSF is present in the signal. This is in line with previous work showing that corrected tensor metrics were more sensitive to tissue alterations compared to non-corrected tensor metrics (Metzler-Baddeley et al., 2012). Collectively, this suggests that correcting for CSF-based contamination is necessary in diffusion MRI studies in HD and performing FWE can alter the statistical outcome and subsequent interpretation of the data, with implications for both past and future studies. Furthermore, this highlights the interaction between tissue macrostructure and tissue microstructure, which is important for HD where alterations may occur in both.

An additional benefit of the CSF correction approach applied here is the creation of free water fraction map, or F, which is the inverse of TVF. Free water fraction has recently been shown to be sensitive to disease progression in Parkinson's disease, predicting changes in bradykinesia and cognition over a 1-year period (Ofori et al., 2015). In this work, we show that TVF is reduced in HD participants and is associated with both disease burden and motor speed, suggesting that the metric may also have utility in understanding HD disease progression. The relationship with disease burden is especially informative in the pre-symptomatic and early disease stages when other clinical predictors (e.g. motor signs) may not be as evident. One benefit of using the disease burden index is the normal distribution of disease burden indices in a HD research cohort, whereas there is a skewed distribution for many of the measures which are also less sensitive to the earlier stages of HD. For example, the TFC scale was originally designed to assess progression of HD in symptomatic patients, whereas the cohort recruited in this study are in the pre-symptomatic and early stages of the disease. The observed relationship between disease burden index and MRI metrics can potentially serve as a biomarker for disease burden, useful for longitudinal studies and assessing the outcome of therapeutic interventions. The benefit of the TVF metric is that a separate acquisition is not required, thus there is no cost associated in order to gain this additional metric. Although TVF provides increased sensitivity in terms of the relationship with clinical symptoms, in terms of biophysical specificity, the observed reduction in TVF along a white matter pathway is suggestive of a reduction in packing density in the tissue, which may be due to a number of factors such as neuronal loss in neighbouring grey matter, and/or white matter atrophy, such as a loss of axons or demyelination.

HD is a rare condition; consequently single-site studies such as this commonly suffer problems with small sample sizes. However, the addition of neuroimaging to international multi-site study protocols (e.g. PREDICT-HD; Paulsen et al., 2014) holds promise for increasing the reliability of results and the scope of data analyses in clinical neuroimaging of HD. Recently, an analysis of the effects of between-site variability in diffusion MRI acquisition in HD showed that multi-site pooling of diffusion MR data can be performed in a valid manner with consistent group differences in FA values (Müller et al., 2013) suggesting that multi-site diffusion MRI analysis is feasible. It is likely that CSF contamination is more of an issue in structures closer to CSF-boundaries, such as the corpus callosum (Jones et al., 2005) and the fornix (Metzler-Baddeley et al., 2012), and in areas of disease-related atrophy. Nevertheless, the post hoc correction used in this study is rapid and applied to the whole brain with little additional cost, and where CSF contamination is not present, the algorithm is likely to have little impact.

In conclusion, this work suggests researchers should be both aware of, and take steps to address, confounds in the analysis and interpretation of diffusion MRI data in HD. The recommendations laid out in this work will lead to more anatomically faithful tract reconstructions through the amelioration of confounds that are particularly problematic in a HD cohort. Performing the correction for CSF contamination was shown to reduce within-group variance with implications for the sample size required to detect an effect, which is an important consideration in the study of a rare disease such as HD. Finally, we recommend TVF as a complementary metric alongside tensor-based metrics, based on the observed increased sensitivity to clinical measures. Taken together, this improved sensitivity and validity of results from diffusion MRI data has the potential to lead to new insights into white matter microstructure and macrostructural alterations in HD if adopted.

## Figures and Tables

**Table 1:** Demographics of HD and control cohort.

	HD (*n* = 15)	Healthy controls (*n* = 13)	*p*-Value
Age	45.5 ± 2.7	46.8 ± 2.9	0.747
Gender	7 M	6 M	0.978
Education (ISCED)	3.9 ± 0.4	3.5 ± 0.4	0.487
CAG	43 ± 0.75	n/a	
Disease burden	330.9 ± 29.3	n/a	
# Manifest	5	n/a	
UHDRS motor	15.8 ± 5.1	n/a	
UHDRS TFC	11.1 ± 0.7	n/a	

Mean and standard error of the mean shown. ISCED: International Standard Classification of Education; UHDRS: United Huntington's Disease Rating Scale; TFC: Total Functional Capacity.

## References

[bib0005] Alexander A., Hasan K.M., Lazar M., Tsuruda J.S., Parker D.L. (2001). Analysis of partial volume effects in diffusion-tensor MRI. Magn Reson Med.

[bib0010] Alexander D. (2005). Multiple-fiber reconstruction algorithms for diffusion MRI. Ann NY Acad Sci.

[bib0015] Andersson J.L.R., Jenkinson M., Smith S. (2010). Non-linear registration, aka spatial normalisation.

[bib0020] Aylward E.H., Nopoulos P.C., Ross C.A., Langbehn D.R., Pierson R.K., Mills J.A. (2011). Longitudinal change in regional brain volumes in prodromal Huntington disease. J Neurol Neurosurg Psychiatry.

[bib0025] Baron C.A., Beaulieu C. (2015). Acquisition strategy to reduce cerebrospinal fluid partial volume effects for improved DTI tractography. Magn Reson Med.

[bib0030] Bartzokis G., Lu P.H., Tishler T.A., Fong S.M., Oluwadara B., Finn J.P. (2007). Myelin breakdown and iron changes in Huntington's disease: pathogenesis and treatment implications. Neurochem Res.

[bib0035] Basser P.J., Mattiello J., LeBihan D. (1994). Estimation of the effective self-diffusion tensor from the NMR spin echo. J Magn Reson B.

[bib0040] Basser P.J., Pajevic S., Pierpaoli C., Duda J., Aldroubi A. (2000). In vivo fiber tractography using DT-MRI data. Magn Reson Med.

[bib0045] Benjamini Y., Hochberg Y. (1995). Controlling the false discovery rate: a practical and powerful approach to multiple testing. J Royal Stat Soc Ser B (Methodol).

[bib0050] Bohanna I., Georgiou-Karistianis N., Sritharan A., Asadi H., Johnston L., Churchyard (2011). Diffusion tensor imaging in Huntington's disease reveals distinct patterns of white matter degeneration associated with motor and cognitive deficits. Brain Imaging Behav.

[bib0055] Brockstedt S., Borg M., Geijer B., Wirestam R., Thomsen C., Holtås S. (1999). Triggering in quantitative diffusion imaging with single-shot EPI. Acta Radiol (Stockholm, Sweden: 1987).

[bib0060] Chang L.-C., Jones D.K., Pierpaoli C. (2005). RESTORE: robust estimation of tensors by outlier rejection. Magn Reson Med.

[bib0065] Chang L.-C., Walker L., Pierpaoli C. (2012). Informed RESTORE: a method for robust estimation of diffusion tensor from low redundancy datasets in the presence of physiological noise artifacts. Magn Reson Med.

[bib0070] Chou M.-C., Lin Y.-R., Huang T.-Y., Wang C.-Y., Chung H.-W., Juan C.-J. (2005). FLAIR diffusion-tensor MR tractography: comparison of fiber tracking with conventional imaging. AJNR Am J Neuroradiol.

[bib0075] Conturo T.E., Lori N.F., Cull T.S., Akbudak E., Snyder A.Z., Shimony J.S. (1999). Tracking neuronal fiber pathways in the living human brain. Proc Natl Acad Sci USA.

[bib0080] Cook P.A., Bai Y., Nedjati-Gilani S., Seunarine K.K., Hall M.G., Parker G.J. (2006). Camino: open-source diffusion – MRI reconstruction and processing. Proc. Annual Meet. ISMRM.

[bib0085] Cook P.A., Symms M., Boulby P.A., Alexander D. (2007). Optimal acquisition orders of diffusion-weighted MRI measurements. J Magn Reson Imaging.

[bib0090] Dell’Acqua F., Scifo P., Rizzo G., Catani M., Simmons A., Scotti G. (2010). A modified damped Richardson–Lucy algorithm to reduce isotropic background effects in spherical deconvolution. Neuroimage.

[bib0095] Di Paola M., Luders E., Cherubini A., Sanchez-Castaneda C., Thompson P.M., Toga A.W. (2012). Multimodal MRI analysis of the corpus callosum reveals white matter differences in presymptomatic and early Huntington's disease. Cereb Cortex.

[bib0100] Di Paola M., Phillips O.R., Sanchez-Castaneda C., Di Pardo A., Maglione V., Caltagirone C. (2014). MRI measures of corpus callosum iron and myelin in early Huntington's disease. Hum Brain Mapp.

[bib0105] Dietschy J.M., Turley S.D. (2004). Thematic review series: brain lipids. Cholesterol metabolism in the central nervous system during early development and in the mature animal. J Lipid Res.

[bib0115] Dumas E.M., van den Bogaard S.J.A., Ruber M.E., Reilman R.R., Stout J.C., Craufurd D. (2012). Early changes in white matter pathways of the sensorimotor cortex in premanifest Huntington's disease. Hum Brain Mapp.

[bib0120] Frank L. (2002). Characterization of anisotropy in high angular resolution diffusion-weighted MRI. Magn Reson Med.

[bib0125] Greenhouse S.W., Geisser S. (1959). On methods in the analysis of profile data. Psychometrika.

[bib0130] Habib J., Hlinka J., Sotiropoulos S., Tench C., Auer D., Morgan P. (2008). Relevance of cardiac-gating in longitudinal diffusion weighted MRI studies. Proc. Annual Meet. ISMRM.

[bib0135] Habib J., Auer D., Morgan P. (2009). Do we need cardiac gating in brain-DTI at high (3T) and ultra-high (7T) field strengths?. Proc. Annual Meet. ISMRM.

[bib0140] Henkelman R.M. (1985). Measurement of signal intensities in the presence of noise in MR images. Med Phys.

[bib0145] Hofer S., Frahm J. (2006). Topography of the human corpus callosum revisited – comprehensive fiber tractography using diffusion tensor magnetic resonance imaging. Neuroimage.

[bib0150] Jeurissen B., Leemans A., Tournier J.-D., Jones D.K., Sijbers J. (2013). Investigating the prevalence of complex fiber configurations in white matter tissue with diffusion magnetic resonance imaging. Hum Brain Mapp.

[bib0155] Jones D.K. (2008). Studying connections in the living human brain with diffusion MRI. Cortex.

[bib0160] Jones D.K. (2010). Precision and accuracy in diffusion tensor magnetic resonance imaging. Top Magn Reson Imaging.

[bib0165] Jones D.K., Cercignani M. (2010). Twenty-five pitfalls in the analysis of diffusion MRI data. NMR Biomed.

[bib0170] Jones D.K., Horsfield M.A., Simmons A. (1999). Optimal strategies for measuring diffusion in anisotropic systems by magnetic resonance imaging. Magn Reson Med.

[bib0175] Jones D.K., Leemans A. (2011). Diffusion tensor imaging. Methods Mol Biol.

[bib0180] Jones D.K., Pierpaoli C. (2005). Contribution of cardiac pulsation to variability of tractography results. Proc. Annual Meet. ISMRM.

[bib0185] Jones D.K., Simmons A., Williams S.C., Horsfield M.A. (1999). Non-invasive assessment of axonal fiber connectivity in the human brain via diffusion tensor MRI. Magn Reson Med.

[bib0190] Jones D.K., Travis A.R., Eden G., Pierpaoli C., Basser P.J. (2005). PASTA: pointwise assessment of streamline tractography attributes. Magn Reson Med.

[bib0195] Kincses Z.T., Szabó N., Tóth E., Zádori D., Faragó P., Németh D. (2013). Diffusion MRI measured white matter microstructure as a biomarker of neurodegeneration in preclinical Huntington's disease. Ideggyógy Szle.

[bib0200] Klein S., Staring M., Murphy K., Viergever M.A., Pluim J.P. (2010). elastix: a toolbox for intensity-based medical image registration. IEEE Trans Med Imaging.

[bib0205] Le Bé J.-V., Silberberg G., Wang Y., Markram H. (2007). Morphological, electrophysiological, and synaptic properties of corticocallosal pyramidal cells in the neonatal rat neocortex. Cereb Cortex.

[bib0210] Le Bihan D., Poupon C., Amadon A., Lethimonnier F. (2006). Artifacts and pitfalls in diffusion MRI. J Magn Reson Imaging.

[bib0215] Leemans A., Jeurissen B., Sijbers J. (2009). ExploreDTI: a graphical toolbox for processing, analyzing, and visualizing diffusion MR data. Proc. Annual Meet. ISMRM.

[bib0220] Leemans A., Jones D.K. (2009). The B-matrix must be rotated when correcting for subject motion in DTI data. Magn Reson Med.

[bib0225] Li H., Li S.H., Yu Z.X., Shelbourne P., Li X.J. (2001). Huntingtin aggregate-associated axonal degeneration is an early pathological event in Huntington's disease mice. J Neurosci.

[bib0230] Li J.Y., Conforti L. (2013). Axonopathy in Huntington's disease. Exp Neurol.

[bib0235] Li X.J. (1999). The early cellular pathology of Huntington's disease. Mol Neurobiol.

[bib0240] Magnotta V., Kim J., Koscik T., Beglinger L., Espinso D., Langbehn D. (2009). Diffusion tensor imaging in preclinical Huntington's disease. Brain Imaging Behav.

[bib0245] Mangin J.-F., Poupon C., Clark C., Le Bihan D., Bloch I. (2002). Distortion correction and robust tensor estimation for MR diffusion imaging. Med Image Anal.

[bib0250] Metzler-Baddeley C., O'Sullivan M., Bells S., Pasternak O., Jones D.K. (2012). How and how not to correct for CSF-contamination in diffusion MRI. Neuroimage.

[bib0255] Mori S., Crain B.J., Chacko V.P., van Zijl P.C. (1999). Three-dimensional tracking of axonal projections in the brain by magnetic resonance imaging. Ann Neurol.

[bib0260] Mori S., Wakana S., Nagae-Poetscher L., van Zijl P. (2005). MRI atlas of human white matter.

[bib0265] Morris D., Nossin-Manor R., Taylor M.J., Sled J.G. (2011). Preterm neonatal diffusion processing using detection and replacement of outliers prior to resampling. Magn Reson Med.

[bib0270] Müller H.-P., Glauche V., Novak M.J.U., Nguyen-Thanh T., Unrath A., Lahiri N. (2011). Stability of white matter changes related to Huntington's disease in the presence of imaging noise: a DTI study. PLoS Curr.

[bib0275] Müller H.-P., Grön G., Sprengelmeyer R., Kassubek J., Ludolph A.C., Hobbs N. (2013). Evaluating multicenter DTI data in Huntington's disease on site specific effects: an ex post facto approach. Neuroimage Clin.

[bib0280] Nopoulos P., Magnotta V.A., Mikos A., Paulson H., Andreasen N.C., Paulsen J.S. (2007). Morphology of the cerebral cortex in preclinical Huntington's disease. Am J Psychiatry.

[bib0285] O’Donnell L.J., Pasternak O. (2014). Does diffusion MRI tell us anything about the white matter? An overview of methods and pitfalls. Schizophr Res.

[bib0290] Ofori E., Pasternak O., Planetta P.J., Burciu R., Snyder A.Z., Febo M. (2015). Increased free water in the substantia nigra of Parkinson's disease: a single-site and multi-site study. Neurobiol Aging.

[bib0295] Papadakis N.G., Martin K.M., Mustafa M.H., Wilkinson I.D., Griffiths P.D., Huang C.L.-H. (2002). Study of the effect of CSF suppression on white matter diffusion anisotropy mapping of healthy human brain. Magn Reson Med.

[bib0300] Pasternak O., Koerte I.K., Bouix S., Fredman E., Sasaki T., Mayinger M. (2014). Hockey Concussion Education Project, Part 2. Microstructural white matter alterations in acutely concussed ice hockey players: a longitudinal free-water MRI study. J Neurosurg.

[bib0305] Pasternak O., Sochen N., Gur Y., Intrator N., Assaf Y. (2009). Free water elimination and mapping from diffusion MRI. Magn Reson Med.

[bib0310] Pasternak O., Westin C.-F., Bouix S., Seidman L.J., Goldstein J.M., Woo T.-U.W. (2012). Excessive extracellular volume reveals a neurodegenerative pattern in schizophrenia onset. J Neurosci.

[bib0315] Paulsen J.S., Hayden M., Stout J.C., Langbehn D.R., Aylward E., Ross C.A. (2006). Preparing for preventive clinical trials: the predict-HD study. Arch Neurol.

[bib0320] Paulsen J.S., Nopoulos P.C., Aylward E., Ross C.A., Johnson H., Magnotta V.A. (2010). Striatal and white matter predictors of estimated diagnosis for Huntington disease. Brain Res Bull.

[bib0325] Paulsen J.S., Long J.D., Ross C.A., Harrington D.L., Erwin C.J., Williams J.K. (2014). Prediction of manifest Huntington's disease with clinical and imaging measures: a prospective observational study. Lancet Neurol.

[bib0330] Penney J.B., Vonsattel J.P., MacDonald M.E., Gusella J.F., Myers R.H. (1997). CAG repeat number governs the development rate of pathology in Huntington's disease. Ann Neurol.

[bib0335] Phillips O., Sanchez-Castaneda C., Elifani F., Maglione V., Di Pardo A., Caltagirone C. (2013). Tractography of the corpus callosum in Huntington's disease. PloS One.

[bib0340] Phillips O., Squitieri F., Sanchez-Castaneda C., Elifani F., Griguoli A., Maglione V. (2014). The corticospinal tract in Huntington's disease. Cereb Cortex.

[bib0345] Pierpaoli C., Jones D.K. (2004). Removing CSF contamination in brain DT-MRIs by using a two-compartment tensor model. Proc. Annual Meet. ISMRM.

[bib0350] Poudel G.R., Stout J.C., Domínguez D.J.F., Salmon L., Churchyard A., Chua P. (2014). White matter connectivity reflects clinical and cognitive status in Huntington's disease. Neurobiol Dis.

[bib0355] Poudel G.R., Stout J.C., Domínguez D.J.F., Churchyard A., Chua P., Egan G.F. (2015). Longitudinal change in white matter microstructure in Huntington's disease: the IMAGE-HD study. Neurobiol Dis.

[bib0360] Reitan R.M. (1979). Manual for administration and scoring of the Halstead–Reitan of neuropsychological test battery for adults and children.

[bib0365] Rosas H.D., Lee S.Y., Bender A.C., Zaleta A.K., Vangel M., Yu P. (2010). Altered white matter microstructure in the corpus callosum in Huntington's disease: implications for cortical “disconnection”. Neuroimage.

[bib0370] Rosas H.D., Reuter M., Doros G., Lee S.Y., Triggs T., Malarick K. (2011). A tale of two factors: what determines the rate of progression in Huntington's disease? A longitudinal MRI study. Mov Disord.

[bib0375] Rosas H.D., Salat D.H., Lee S.Y., Zaleta A.K., Pappu V., Fischl B. (2008). Cerebral cortex and the clinical expression of Huntington's disease: complexity and heterogeneity. Brain.

[bib0380] Sach M., Winkler G., Glauche V., Liepert J., Heimbach B., Koch M.A. (2004). Diffusion tensor MRI of early upper motor neuron involvement in amyotrophic lateral sclerosis. Brain.

[bib0385] Saher G., Brügger B., Lappe-Siefke C., Möbius W., Tozawa R., Wehr M.C. (2005). High cholesterol level is essential for myelin membrane growth. Nat Neurosci.

[bib0390] Shoulson I., Fahn S. (1979). Huntington disease: clinical care and evaluation. Neurology.

[bib0395] Sinadinos C., Burbidge-King T., Soh D., Thompson L.M., Marsh J.L., Wyttenbach A. (2009). Live axonal transport disruption by mutant Huntingtin fragments in Drosophila motor neuron axons. Neurobiol Dis.

[bib0400] Stern Y. (2009). Cognitive reserve. Neuropsychologia.

[bib0405] Tabrizi S.J., Reilmann R., Roos R.A.C., Durr A., Leavitt B., Owen G. (2012). Potential endpoints for clinical trials in premanifest and early Huntington's disease in the TRACK-HD study: analysis of 24 month observational data. Lancet Neurol.

[bib0410] Tabrizi S.J., Scahill R.I., Durr A., Roos R.A., Leavitt B.R., Jones R. (2011). Biological and clinical changes in premanifest and early stage Huntington's disease in the TRACK-HD study: the 12-month longitudinal analysis. Lancet Neurol.

[bib0415] Tournier J.D., Calamante F., Connelly A. (2007). Robust determination of the fibre orientation distribution in diffusion MRI: non-negativity constrained super-resolved spherical deconvolution. Neuroimage.

[bib0420] Tournier J.D., Mori S., Leemans A. (2011). Diffusion tensor imaging and beyond. Magn Reson Med.

[bib0425] Tuch D.S., Reese T.G., Wiegell M.R., Makris N., Belliveau J.W., Wedeen V.J. (2002). High angular resolution diffusion imaging reveals intravoxel white matter fiber heterogeneity. Magn Reson Med.

[bib0430] Valenza M., Carroll J.B., Leoni V., Bertram L.N., Björkhem I., Singaraja R.R. (2007). Cholesterol biosynthesis pathway is disturbed in YAC128 mice and is modulated by Huntingtin mutation. Hum Mol Genet.

[bib0435] Valenza M., Cattaneo E. (2010). Neuroprotection and brain cholesterol biosynthesis in Huntington's disease. Proc Natl Acad Sci USA.

[bib0440] Valenza M., Rigamonti D., Goffredo D., Zuccato C., Fenu S., Jamot L. (2005). Dysfunction of the cholesterol biosynthetic pathway in Huntington's disease. J Neurosci.

[bib0445] Vonsattel J.P., Myers R.H., Stevens T.J., Ferrante R.J., Bird E.D., Richardson E.P. (1985). Neuropathological classification of Huntington's disease. J Neuropathol Exp Neurol.

[bib0450] Vos S.B., Jones D.K., Viergever M.A., Leemans A. (2011). Partial volume effect as a hidden covariate in DTI analyses. Neuroimage.

[bib0455] Walker L., Chang L.-C., Koay C.G., Sharma N., Cohen L., Verma R. (2011). Effects of physiological noise in population analysis of diffusion tensor MRI data. Neuroimage.

[bib0460] Wang Y., Wang Q., Haldar J.P., Yeh F.-C., Xie M., Sun P. (2011). Quantification of increased cellularity during inflammatory demyelination. Brain.

[bib0465] Wedeen V.J., Hagmann P., Tseng W.-Y.I., Reese T.G., Weisskoff R.M. (2005). Mapping complex tissue architecture with diffusion spectrum magnetic resonance imaging. Magn Reson Med.

[bib0470] Wu M., Chang L.C., Walker L., Lemaitre H., Barnett A.S., Marenco S. (2008). Comparison of EPI distortion correction methods in diffusion tensor MRI using a novel framework. Med Image Comput Comput Assist Interven.

[bib0475] Xiang Z., Valenza M., Cui L., Leoni V., Jeong H.-K., Brilli E. (2011). Peroxisome-proliferator-activated receptor gamma coactivator 1α contributes to dysmyelination in experimental models of Huntington's disease. J Neurosci.

[bib0480] Zhou Z., Liu W., Cui J., Wang X., Arias D., Wen Y. (2011). Automated artifact detection and removal for improved tensor estimation in motion-corrupted DTI data sets using the combination of local binary patterns and 2D partial least squares. J Magn Reson Imaging.

[bib0485] Zwiers M.P. (2010). Patching cardiac and head motion artefacts in diffusion-weighted images. Neuroimage.

