# Supplementary material for: Robust MR-based approaches to quantifying white matter structure and structure/function alterations in Huntington's disease
Source: J Neurosci Methods. 2016 May 30;265:2–12. doi: 10.1016/j.jneumeth.2015.08.027 (PMC4863525; doi:10.1016/j.jneumeth.2015.08.027)
Supplement: Supplementary file 1 [file mmc1.docx]

Robust MR-based approaches to quantifying white matter structure and structure/function alterations in Huntington’s disease

Steventon et al, 2015

**Supplementary information**

*Estimation of the diffusion tensor and outlier rejection*

Artefactual data points were identified using a modified version of the robust estimation of tensors by outlier rejection (RESTORE) algorithm (Chang et al., 2005). The threshold criterion implemented in Chang et al. (2005) was used (σ = 1.5267 * standard deviation of the background noise) (Henkelman, 1985) to assess the data fit. For this dataset the mean σ was 87.67 ± 13.65 SD, with no difference in the standard deviation of background noise between HD and control participants (p>0.05). The mean percentage of artefactual data points identified per dataset across the whole brain was 0.59 % (SD: 0.19 %) with significantly more data points rejected for HD datasets (0.65 ± 0.21 %) compared to control datasets (0.51 ±0.16 %), t_25_= -2.20, p < 0.05 (see Supplementary Figure 1 for spatial distribution of outliers).

Table 1 diffusion parameters for the twice-refocused spin echo (TRSE) sequences used, with onset time for diffusion gradients of length δ (ms).


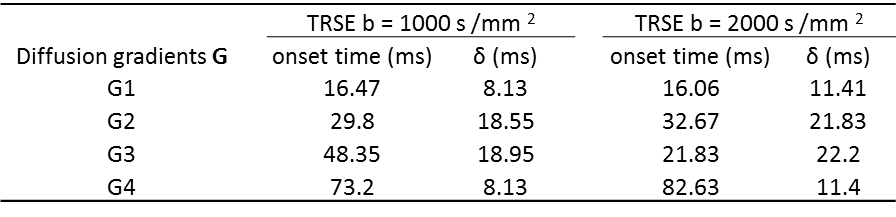


Table S2. Statistics from post-hoc analyses of group-based differences (hd vs control). free water correction affects the statistical outcome differentially depending on the segment, metric and analysis (roi vs tractography). ↑ indicates an increase in the q-value and ↓ indicates a decrease in the q-value

| **Metric** | **Segment of interest** | **Free-water corrected?** | **F statistic** | **Uncorrected p-value** | **FDR-adjusted q value** | **∆ q-value after correction** |
| --- | --- | --- | --- | --- | --- | --- |
| **ROIs** | |  |  |  |  |  |
| FA | GCC | No | 11.34 | 0.002 | **0.006** | ↑ |
|  |  | Yes | 9.05 | 0.006 | **0.009** |  |
|  | BCC | No | 11.03 | 0.003 | **0.007** | ↑ |
|  |  | Yes | 9.57 | 0.005 | **0.009** |  |
|  | SCC | No | 10.28 | 0.004 | **0.008** | ↑ |
|  |  | Yes | 7.19 | 0.013 | **0.016** |  |
| MD | GCC | No | 14.63 | 0.001 | **0.004** | = |
|  |  | Yes | 15.16 | 0.001 | **0.004** |  |
|  | BCC | No | 7.15 | 0.013 | **0.016** | ↑ |
|  |  | Yes | 5.23 | 0.029 | **0.029** |  |
|  | SCC | No | 13.01 | 0.001 | **0.004** | ↑ |
|  |  | Yes | 5.58 | 0.026 | **0.028** |  |
| **Callosal Tracts** | |  |  |  |  |  |
| MD (DTI) | Segment I | No | 4.19 | 0.053 | 0.133 | ↓ |
|  |  | Yes | 5.51 | **0.028** | 0.093 |  |
|  | Segment II | No | 0.78 | 0.387 | 0.455 | ↓ |
|  |  | Yes | 9.55 | **0.005** | **0.040** |  |
|  | Segment III | No | 0.78 | 0.387 | 0.455 | ↓ |
|  |  | Yes | 1.45 | 0.242 | 0.406 |  |
|  | Segment IV | No | 0.32 | 0.577 | 0.607 | ↓ |
|  |  | Yes | 0.92 | 0.348 | 0.455 |  |
|  | Segment V | No | 10.36 | **0.004** | **0.040** | = |
|  |  | Yes | 9.09 | **0.006** | **0.040** |  |
| MD (SD) | Segment I | No | 3.17 | 0.089 | 0.178 | ↓ |
|  |  | Yes | 6.33 | **0.020** | 0.088 |  |
|  | Segment II | No | 0.89 | 0.357 | 0.455 | ↓ |
|  |  | Yes | 6.06 | **0.022** | 0.088 |  |
|  | Segment III | No | 1.32 | 0.264 | 0.406 | = |
|  |  | Yes | 1.43 | 0.244 | 0.406 |  |
|  | Segment IV | No | 0.01 | 0.931 | 0.931 | ↓ |
|  |  | Yes | 0.45 | 0.511 | 0.568 |  |
|  | Segment V | No | 3.85 | 0.063 | 0.140 | ↓ |
|  |  | Yes | 4.60 | **0.043** | 0.123 |  |

Table S3. mean tissue volume fraction in the reconstructed segments of the corpus callosum reconstructed using both dti- and sd-based tractography. ± standard error of the mean

| **Algorithm** | **Gene status** | **Corpus callosum segment** | | | | |
| --- | --- | --- | --- | --- | --- | --- |
|  |  | **I** | **II** | **III** | **IV** | **V** |
| DTI | Control | 0.67 ± .0063 | 0.64 ± .0186 | .66 ± .0092 | .66 ± .0079 | .68 ± .0049 |
|  | HD | 0.63 ± .0100 | 0.61 ± .0199 | .64 ± .0121 | 0.65 ± .0127 | .63 ± .0304 |
| SD | Control | 0.66 ± .0067 | 0.63 ± .0195 | 0.66 ± .0095 | 0.65 ± .0086 | 0.67 ± .0078 |
|  | HD | 0.62 ± .0113 | 0.59 ± .0213 | 0.63 ± .0106 | 0.65 ± .0106 | 0.62 ± .0294 |


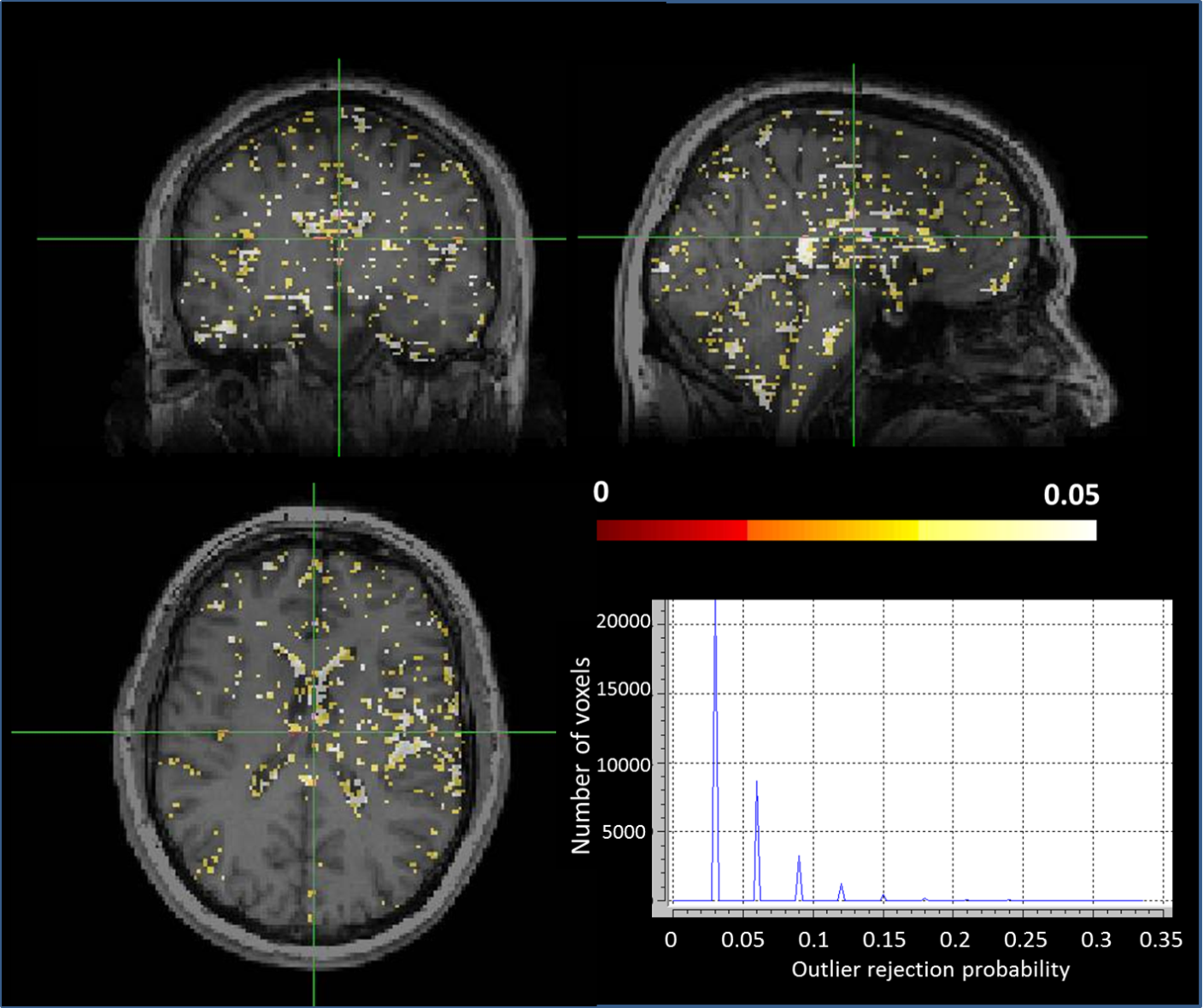


Figure S1. Outlier rejection probability map for a representative dataset with a t1-weighted image overlaid for anatomical reference. Green crosshairs indicate the location of the depicted image slices.


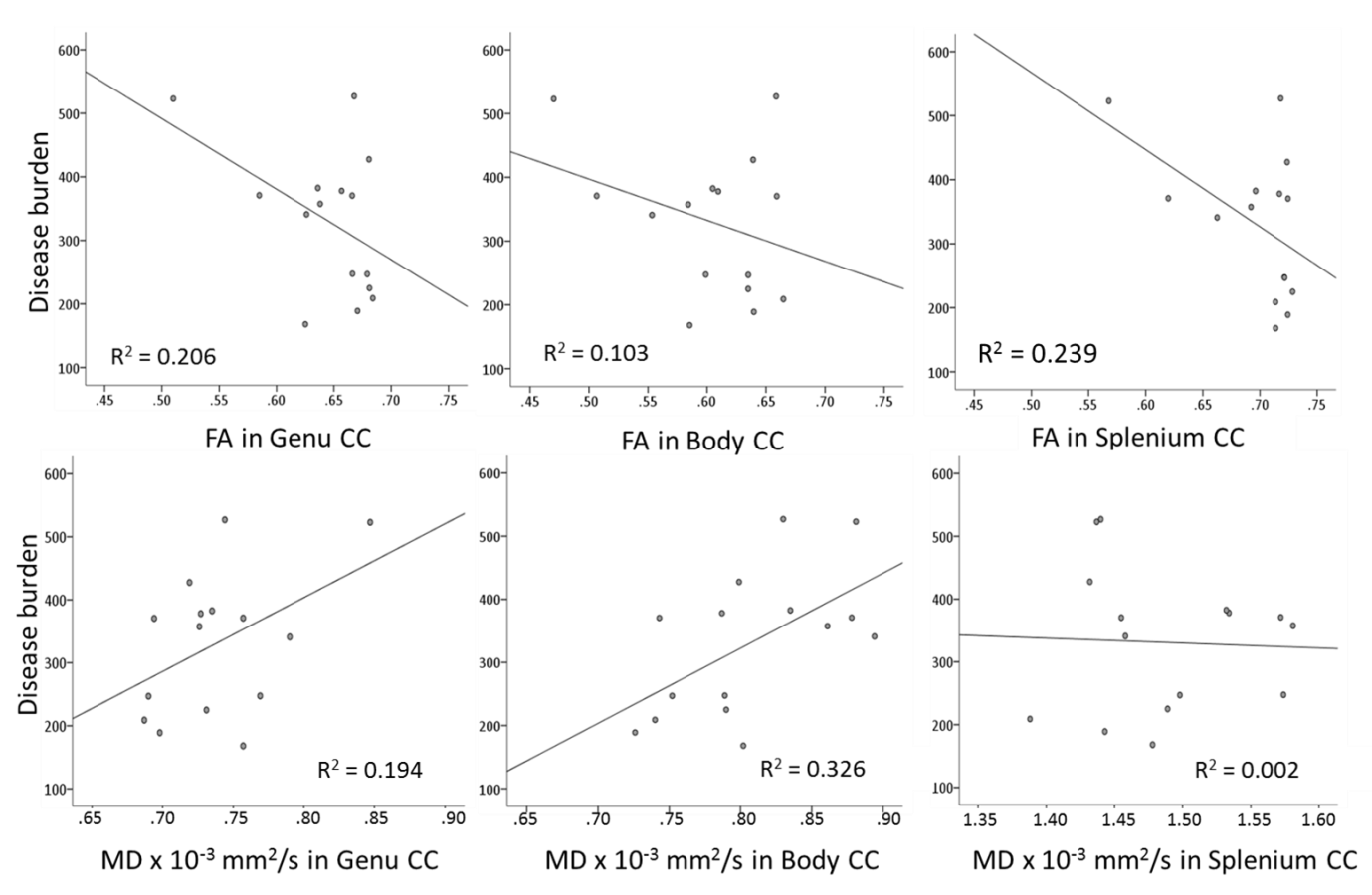


Figure S2. Relationship between disease burden and the diffusion tensor metrics fa and md in the 3 corpus callosum (CC) ROIs with linear fit line overlaid. None of the correlations are significant using the fdr-adjusted p value.
